# The Use of a Tetraminos Chimeric Free Flap in Lower Limb Trauma

**DOI:** 10.7759/cureus.13427

**Published:** 2021-02-18

**Authors:** Timothy Schrire, Asmat H Din, Umraz Khan

**Affiliations:** 1 Plastic Surgery, Southmead Hospital, Bristol, GBR; 2 Plastic and Reconstructive Surgery, Southmead Hospital, Bristol, GBR

**Keywords:** orthoplastic surgery, lower limb trauma, free flap, chimeric flap, lower limb reconstruction

## Abstract

Major trauma care has improved in the UK since the evolution and acceptance of specialist centers . A mission statement for major trauma care is “reduction in mortality and disability following trauma.” The care for extremity trauma has benefited from this specialization. Traumatic loss of skin integument in the extremities, especially over mobile joints, may lead to a compromised functional outcome. Modern reconstructive plastic surgery aims to provide flaps with minimal donor site morbidity.

In this case report, we present the use of two chimeric flaps undertaken sequentially (one acutely and the second delayed) around the knee joint to allow a greater range of motion and function after a severe traumatic event. In this clinical case, the original tissue defects had meant that a free flap was used to reconstruct an open fractured bone, and split skin grafting was undertaken on the anterior aspect of the knee. The latter was then replaced after some months of recovery.

## Introduction

Large soft tissue defects in the lower limb have presented problems for reconstructive surgery as large flaps are needed to optimize function and healing. However, this necessity for tissue is limited by donor site morbidity, as well as the size, shape, and geometric needs of the recipient site. Lower limb joints present a particular issue as they require malleable, dynamic tissue, which can adjust to the stressors placed upon it by the movements of the limb. The use of split skin grafts to cover large defects remains widespread and provides good tissue coverage, but can lead to contractures and reduction in function over joint spaces. This is particularly problematic over joint lines.

There has been use of latissimus dorsi, rectus abdominis, and anterolateral thigh perforator flaps to cover large defects. Some surgeons have advocated the use of chimeric latissimus dorsi-serratus anterior flap reconstruction to provide reliable soft tissue coverage, with greater flexibility of size and geometric pattern [1]. For smaller defects, a medial sural artery perforator flap with a separated piece of gastrocnemius muscle has been shown to provide good three-dimensional reconstructive capabilities in the lower limb [2]. Otherwise, chimeric reconstructions seem to be interspersed between mandibular and upper limb reconstructive surgery [3-5]. This means that in terms of lower limb reconstruction, there is a dearth of information for larger reconstructive free flap options.

There is one case report of a quad flap using the novel combination of scapular, parascapular, latissimus dorsi, and serratus anterior flap to crush injuries in the lower limbs [6]. This complex flap demonstrated excellent tissue coverage, and, in both cases, resulted in an avoidance of amputation and satisfactory functional outcomes. Both of these were in crush injuries, and were used in the emergency setting. The use of a chimeric parascapular and scapular flap has not been reported in the literature for lower limb reconstruction, nor has there been much investigation into the use of free flaps to improve functionality of previously grafted lower limbs. This report is presented according to consensus-based surgical case report guidelines.

## Case presentation

Patient information

**Figure 1:**
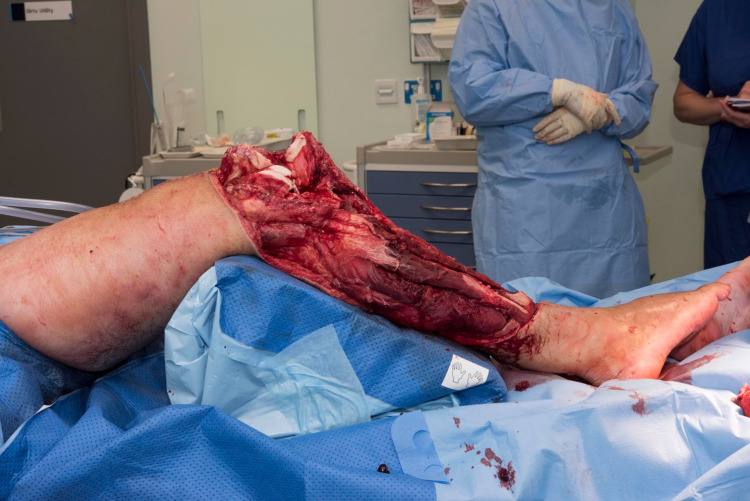
Initial limb defect.

**Figure 2:**
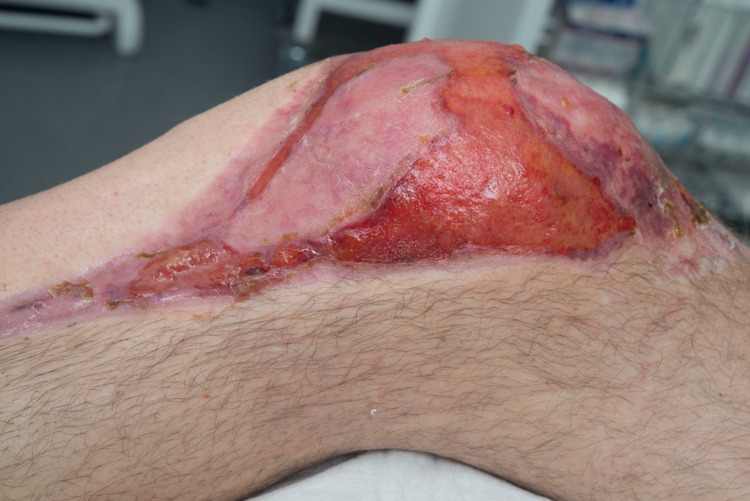
Split skin graft to knee demonstrating tightness and scarring.

**Figure 3:**
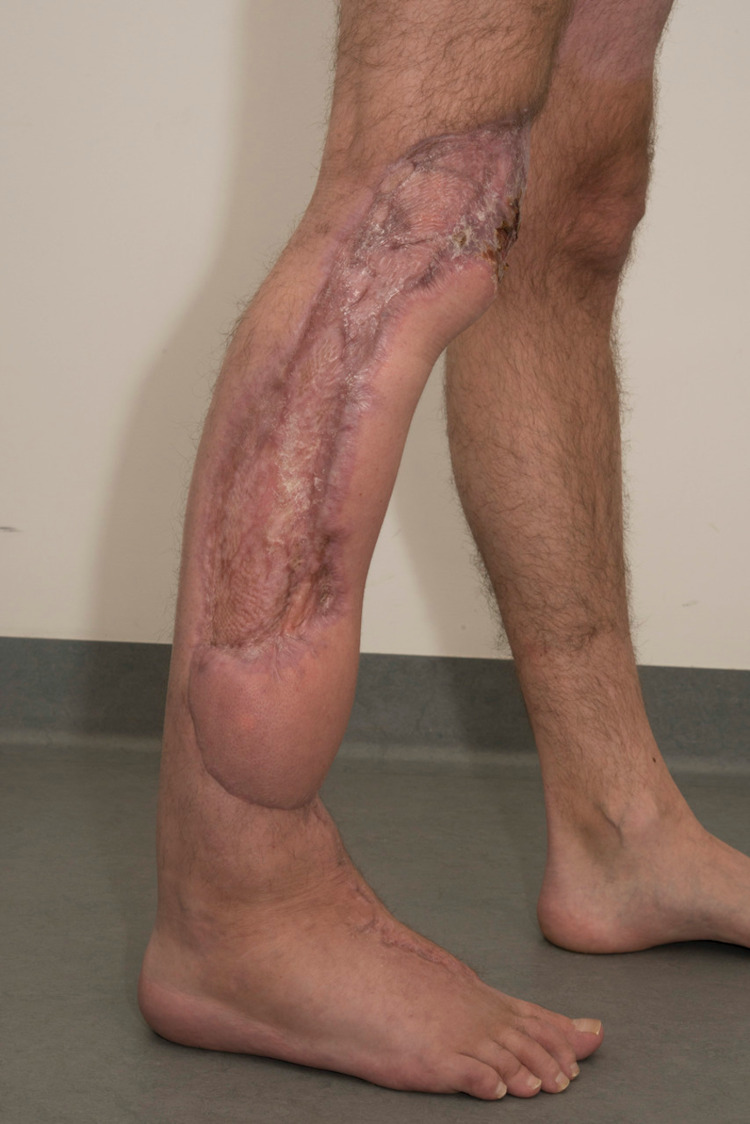
Demonstration of restricted range of motion at knee.

The patient was a 30-year-old male who had been hit at 90 mph while on a motorbike sustaining femoral, tibial, and right foot open fractures. The tissue loss and zone of injury extended from his distal thigh down to the ankle (Figure 1). After assessment by a number of orthopedic and plastics consultants, it was decided to salvage the limb. He underwent an open reduction and internal fixation (ORIF) of his right lateral femoral condyle, a right retrograde femoral nail, and ORIF of the tibia. He had two separate free flaps (a chimera scapular/parascapular and anterolateral thigh) to his right lower leg, and split skin grafting to the remainder of the right lower limb. As the initial defect was too large to be primarily reconstructed with free flaps, coverage of the exposed bone and metalwork was prioritized. He was affected by post-traumatic stress disorder subsequently, along with some chronic pain issues, for which he self-medicated with cannabis oil. He was a smoker of seven a day, but did not take any regular prescribed medications and did not drink. Upon regular post-operative review, it was found that while healed, the limb exhibited a poor return of function, and a major contributor was the lack of motion of the right knee joint (Figures 2, 3).

Clinical findings, diagnosis assessment, and therapeutic intervention

**Figure 4:**
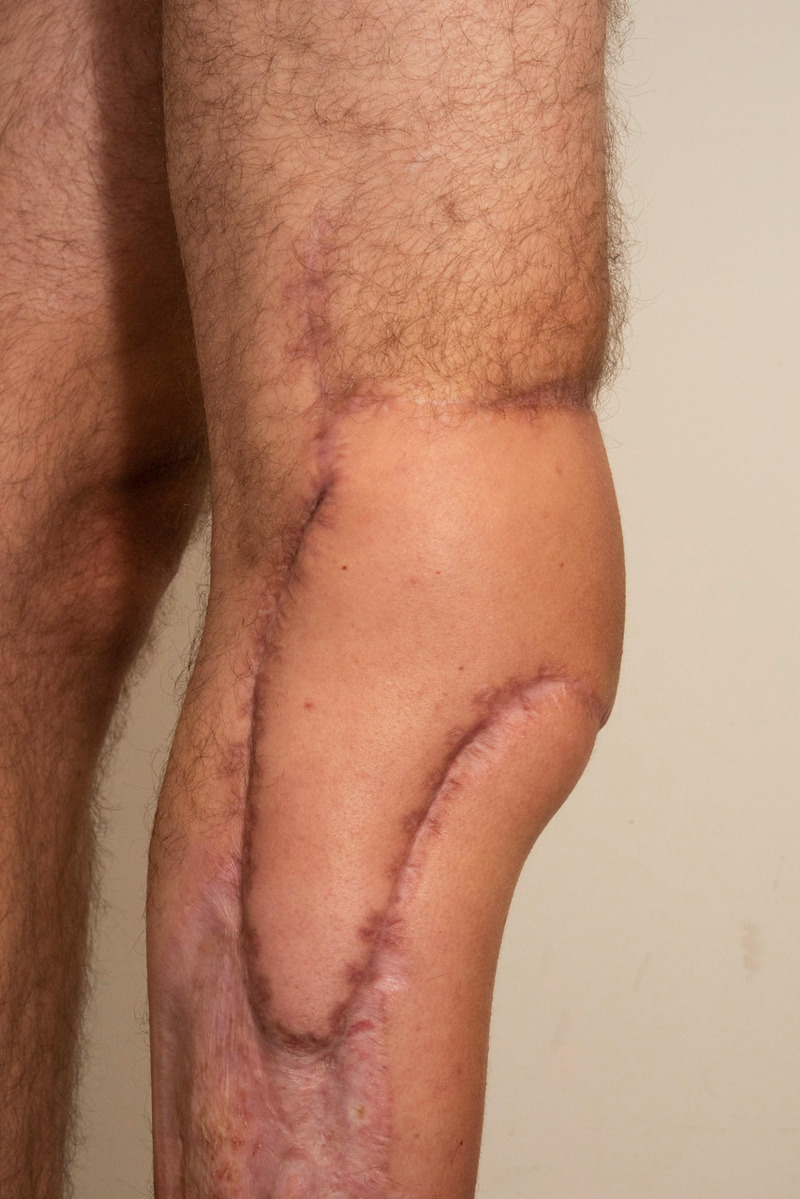
Tetraminos flap—lateral view.

**Figure 5:**
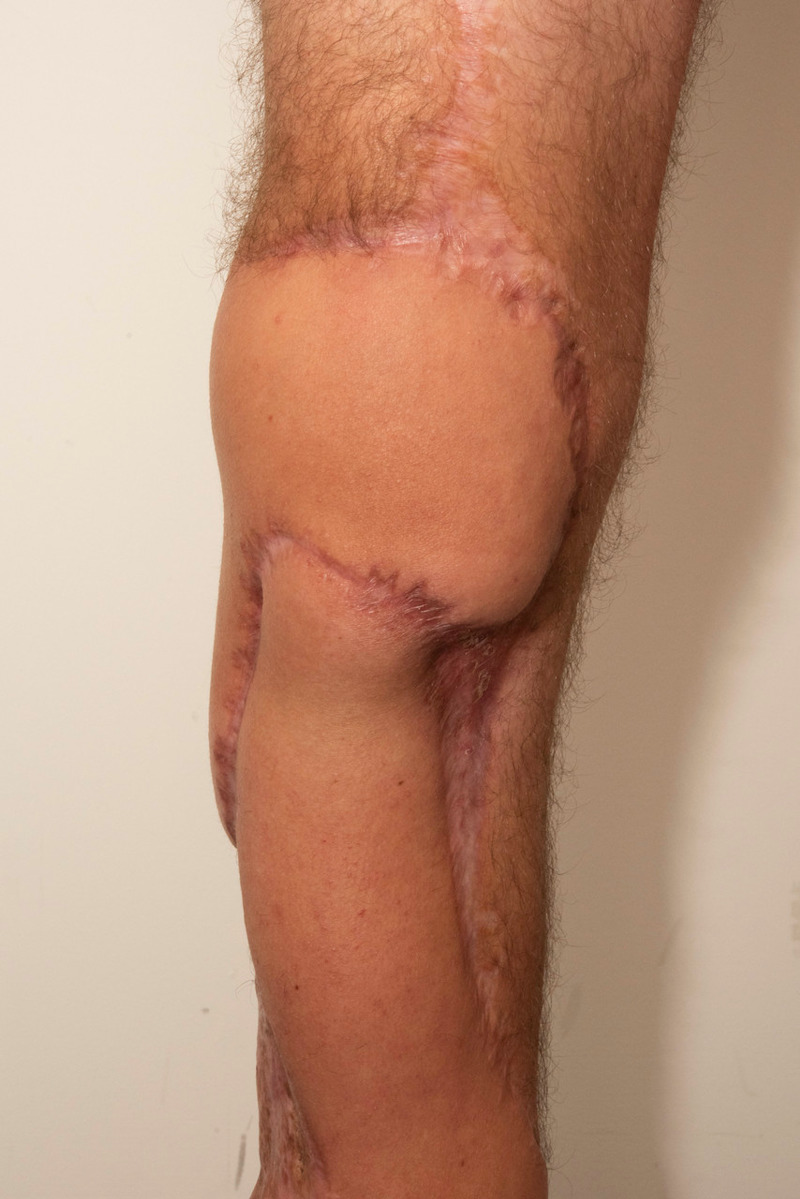
Tetraminos flap—anterior view.

**Figure 6:**
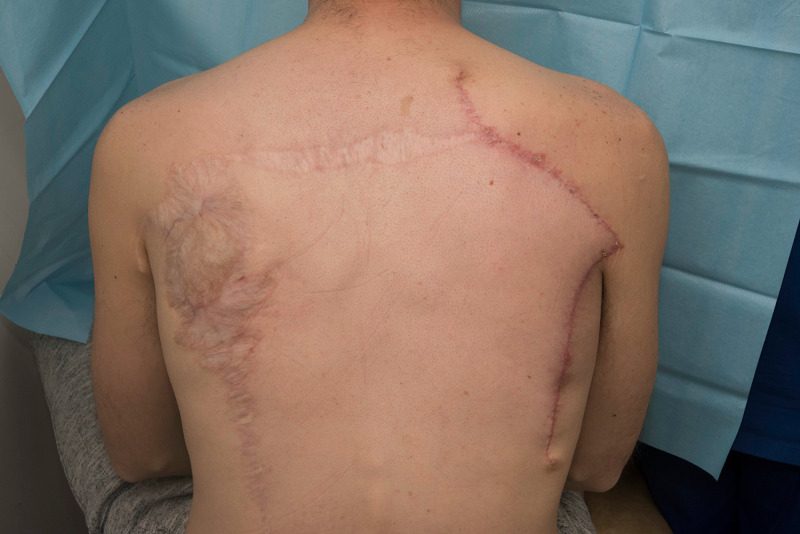
Scapular-parascapular free flap donor site.

The patient was readmitted electively for revisional surgery due to decreased range of motion and functionality in the right knee. He had a concurrent foot drop; however, his limited range of motion, 0-20 degrees, meant that he struggled with stairs, and his general mobilization had to be done with crutches. The split skin graft covered the entire knee joint and extended down across the anterior leg. The plan enacted was an excision of the split skin graft, with a quadriceps plasty, where the myofascial planes were released and the contractures incised, with a chimeric scapular and parascapular free flap onto the defect (Figures 4, 5). End-to-end anastomosis was completed with the lateral genicular artery and concomitant veins. The donor site was closed directly (Figure 6). The patient was transferred to the Plastic Surgery ward, with flap observations according to the protocol [7].

Follow-up and outcome

The patient had an extension block knee brace fitted on day four with a neutral to 35 degree range of motion. This was changed to a removable above knee cast with the ankle flexed at 90 degrees. The patient was mobilized with physiotherapy. Initial dangling resulted in some purpuric discoloration adjacent to the distal flap; however, this subsequently settled and the patient continued to mobilize well with pulpit frame.

**Figure 7:**
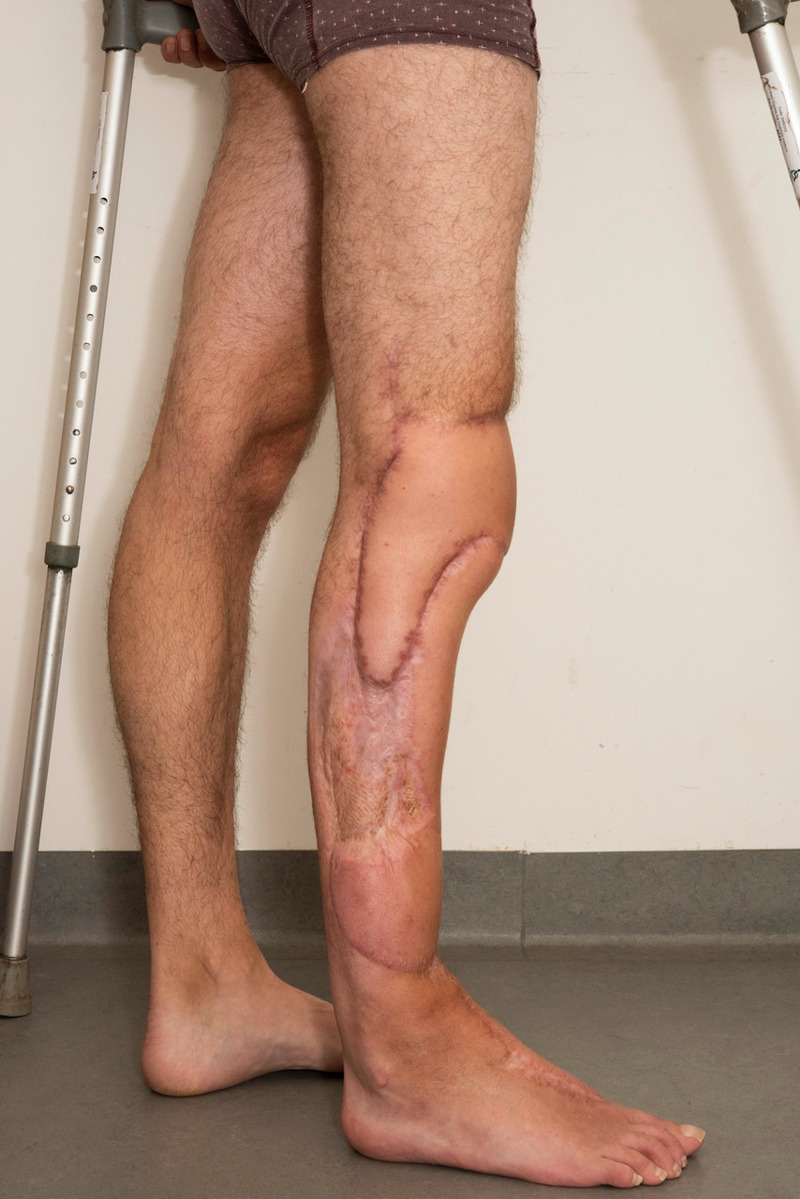
Tetraminos flap reconstruction in use during mobilization.

The range of motion at the time of the operation improved from 20 to 40/50 degrees. This continued to be reflected in the physiotherapy, where the patient continued to strengthen this enhanced range of motion. The flap was reviewed in the clinic one week after discharge, which demonstrated gradual improvement. The patient proceeded to full weight bearing with ongoing physiotherapy after this point and full soft tissue coverage, which no longer restricted his range of motion (Figure 7).

Patient perspective

The patient was aware of the potential risks and benefits of this reconstruction. He was fully engaged in the decision making process, and was an active participant in all aspects of his care. He was compliant during his stay, stopping smoking and adhering strictly to any instructions provided. He was very pleased with the resultant improved function in his range of motion.

## Discussion

Chimeric free flaps have been demonstrated in case reports to provide good coverage in crush injuries to the lower limb. Two cases were reported by Azouz et al. [6] where they used a chimeric latissimus dorsi, serratus anterior, parascapular, and scapular flap in two lower limb reconstructions with significant soft tissue loss. The resultant function reported was satisfactory; however, the aim in these cases was for preservation of the limb, rather than a specific focus on the improvement of lower limb function. The use of free flaps to improve joint range of motion and functionality is, therefore, somewhat novel.

The theory behind the procedure was that by resecting the tight split skin graft and freeing up the myofascial planes with a flexible and dynamic soft tissue coverage would allow greater flexibility in the joint, while additionally providing greater protection to the underlying joint. The chimeric free flap used has demonstrated good effect and improvement in this case; however, a greater number of case reports are needed before any recommendations can be provided. This presents a new, viable option for patients who require improved functionality with a dependable flap over the crucial joints of the lower limb.

## Conclusions

A chimeric flap provides a new alternative for large areas requiring a free flap. The soft tissue coverage achieved can be extensive, with minimal donor site morbidity. This kind of pattern can additionally be used over areas traditionally difficult to cover, such as the knee joint, with the shape allowing inherent flexibility for the recipient site. More studies need to be performed to fully analyze the potential of this kind of flap as its versatility and unique shape will allow multiple applications. Therefore, this represents the first case where the focus was primarily on functional improvement and using solely a parascapular and scapular chimeric flap. While the aesthetic improvement is evident, the functional aspect was illustrated with a doubling of the range of movement.
